# Clinical evaluation of the golden beam data in the monaco treatment planning system for the harmony pro and infinity linear accelerators

**DOI:** 10.1002/acm2.70457

**Published:** 2026-01-05

**Authors:** Wenchao Gao, Min Zhang, Shuchao Zhang, Jie Qi, Xin Wang, Liang Zhao, Ping Liu, Zhijun Yang, HongXiang Cheng

**Affiliations:** ^1^ Department of Radiotherapy Peking University People's Hospital Beijing China; ^2^ Medical Affairs Elekta Instrument (Shanghai) Ltd., Beijing Branch Beijing China

**Keywords:** beam modeling, conventional fractionated radiotherapy, golden beam data, linear accelerator, plan verification, stereotactic body radiotherapy, treatment planning system

## Abstract

**Background:**

Accurate dose calculations in radiotherapy depend on high‐quality beam models in the Monaco treatment planning system (TPS). The accelerated go live (AGL) workflow, using a golden beam data (GBD) model, has improved beam modeling accuracy for various linear accelerators (linacs). However, similar studies specifically for the Harmony Pro, recently introduced for online adaptive radiotherapy, have not yet been reported internationally. Moreover, studies on dosimetric differences between TPS beam models with GBD and those with measured beam data (MBD) are limited, and no such studies have been published specifically for Elekta linacs.

**Purpose:**

This study aimed to assess the clinical performance of GBD in the Monaco TPS for the harmony pro and infinity linacs.

**Methods:**

Beam tuning and data collection were performed on the harmony pro and infinity linacs based on GBD. Subsequently, percentage depth doses (PDDs), off‐axis dose profiles, output factors (OFs), absolute doses, and test fields were measured to evaluate the GBD model. Additionally, 31 clinical plans from multiple anatomical sites, including 17 conventional fractionated radiotherapy (CFRT) plans and 14 stereotactic body radiotherapy (SBRT) plans, were designed using the Monaco TPS (GBD model) and practically tested. An Infinity linac with MBD was introduced as a control.

**Results:**

PDDs and profiles on both GBD linacs showed 100% passing rate (2%/2 mm). OFs and absolute doses on both GBD linacs agreed within ±1% and ±1.5%, respectively. Additionally, verification of the test fields yielded passing rates above 98% (2%/2 mm) for both GBD linacs. Furthermore, for CFRT plans, measurements on three linacs achieved a passing rate above 95% (3%/2 mm). The absolute dose deviations were within 3%, whereas one MBD linac case exceeded 3% (‐3.73%). For SBRT plans, the gamma passing rates were 98.18 ± 1.58%, 98.76 ± 1.54%, and 94.72 ± 0.04% (3%/2 mm) and 96.69 ± 1.96, 96.29 ± 2.26, and 89.51 ± 0.06% (2%/2 mm), for the two GDB linacs and the MDB linac, respectively. The absolute dose deviations were within 3%, whereas two MBD linac cases exceeded 3% (−3.51%, −4.50%).

**Conclusions:**

The harmony pro‐GBD and infinity‐GBD linac results demonstrated strong agreement with GBD. Clinical plans designed with Monaco TPS (GBD model) were clinically acceptable when delivered on both GBD linacs. Although the test results of GBD model plans delivered on the Infinity‐MBD linac showed certain differences compared to those on the two GBD linacs, most plans remained acceptable. This indicates that GBD‐based modeling in Monaco TPS offers reliable clinical performance across different linac types.

## 1 INTRODUCTION

The dose calculation accuracy of a treatment planning system (TPS) directly affects the outcome of radiotherapy treatment. Among various factors contributing to this accuracy, the quality of the beam model is a key determinant. Traditionally, beam modeling in the TPS requires collecting beam data such as percent depth doses (PDDs), off‐axis dose profiles, and output factors (OFs) from linear accelerators (linacs). These measured data are subsequently imported into the TPS, where an iterative optimization algorithm is used to match the simulated beam data to the actual measured data as closely as possible, thereby generating a high‐quality TPS beam model. Consequently, the quality of the beam model and the beam data used for modeling directly influence the dose calculation accuracy of the TPS.

Notably, dosimetric parameters differ not only between linacs from different manufacturers but also among different models produced by the same vendor. Such differences can exist even among nominally identical machines of the same model due to variations in installation and beam tuning. Therefore, prior to clinical use, each linac must undergo individual beam tuning and have its own beam data acquired in accordance with the TPS modeling requirements. Under typical conditions, the time required from the installation and commissioning of a linac to its clinical use is approximately eight weeks, a process that is both time‐consuming and labor‐intensive. Nevertheless, the application of beam matching (BM) in recent years has greatly simplified the commissioning and modeling workflow for multiple linacs from the same vendor. A newly installed linac can be matched to the beam data of a previously commissioned unit, allowing the TPS to directly use the existing beam model for dose calculation without creating a new model. This bypasses the traditional beam modeling process within the TPS, thereby expediting clinical deployment. Furthermore, using BM, good agreement between linacs can be ensured, enabling the interchange of treatment plans across multiple units.^1^, ^2^, ^3^, ^4^, ^5^ However, when a newly installed linac directly adopts the beam data of a previously commissioned unit as its reference, the doses calculated by the TPS may still deviate from measurements by more than 5%, even after completing the BM process.^6^, ^7^ This indicates that BM alone is insufficient to fully ensure the dose calculation accuracy of the TPS beam model.

To address this, a rapid acceptance workflow, Accelerated Go Live (AGL), has been developed based on the golden beam data (GBD) model derived from the aggregated data of thousands of linacs. This workflow spans all stages from installation and commissioning to modeling and treatment delivery, considerably enhancing the accuracy and consistency of the beam model by optimizing both data collection and modeling procedures. The AGL process has been widely applied to various linac models, including Synergy, Infinity, and Versa HD. Additionally, the accuracy of the GBD model on these linacs has been extensively validated by a series of studies.^8^, ^9^, ^10^ Nevertheless, similar studies on Harmony Pro, an online adaptive linac introduced in the past two years, have not yet been reported internationally, nor was its beam data directly compared with those of the Infinity linac. Moreover, research evaluating the reliability of GBD has primarily focused on comparisons between beam modeled with different GBD models. However, relevant studies on dosimetric differences between TPS beam models with GBD and those with traditional measured beam data (MBD) are limited, and no such studies have been published specifically for Elekta linacs. Specifically, the extent of dosimetric discrepancies in Monaco TPS plans based on the GBD model remains unclear when delivered on GBD‐modeled versus MBD‐modeled linacs. Moreover, further verification is necessary to evaluate whether plans delivered on MBD‐modeled linacs meet clinical requirements and to fully validate the clinical applicability of the GBD model.

This study was conducted based on two newly installed linear linacs commissioned using GBD, including the first domestic Harmony Pro linac and one Infinity linac. First, the accuracy of the beam models of these two GBD linacs was evaluated by comparing measured data with GBD model data. Then, clinical conventional fractionation radiotherapy (CFRT) and stereotactic body radiotherapy (SBRT) cases were selected and treatment plans designed in the Monaco TPS based on the GBD model. To compare dosimetric differences between linacs commissioned using GBD and MBD, an Infinity linac based on MBD was introduced as a control. The same plans were executed and measured on the GBD‐ and MBD‐modeled linacs, respectively. The dosimetric differences among the three linacs were compared using parameters such as gamma passing rate and absolute dose deviation to evaluate whether the linacs meet clinical requirements. The accuracy and reliability of the GBD model within the Monaco TPS was thus evaluated.

## 2 MATERIALS AND METHODS

### 2.1 Equipment

In this study, three different linac models were employed for testing, namely Harmony Pro with GDB and Infinity with GDB and MDB (ElektaAB, Stockholm, Sweden). Three machines were equipped with the Agility head, featuring 80 multileaf collimator pairs with 5 mm width. BM between the two linacs was performed using the AGL workflow, and in both cases, the GBD model was employed as the beam model. Another Infinity linac was beam‐commissioned using conventional methods, and its TPS physical model was independently modeled based on collected beam data. Based on clinical requirements, model evaluation was conducted using 6 MV, 6 MV flattening‐filter‐free (FFF) and 10 MV photon beams on each linac.

### 2.2 Beam model verification

Following beam tuning, beam data from both GBD linacs were acquired to verify their agreement with the GBD model. Measurements included PDDs, profiles, OFs, absolute doses, and test fields, as shown in Table 1. PDD, profile, and OF measurements were conducted in a three‐dimensional water tank and compared with the reference data used by the GBD model. For field sizes above 5 × 5 cm, a CC13 ionization chamber (IBA Dosimetry, Schwarzenbruck, Germany) was used for measurements. Alternatively, for the 3 × 3 cm field, a PFD diode detector (IBA Dosimetry, Schwarzenbruck, Germany) was used. Comparisons of PDDs and profiles were performed using the Monaco Commissioning Utility (MCU) with a 2%/2 mm gamma criterion. OFs were normalized to the 10 × 10 cm reference field. Absolute doses were measured in a one‐dimensional water tank and compared with the values calculated by the TPS using the GBD model. Test field measurements, encompassing oval (OAVL), C‐shaped (C), and T‐shaped (T) fields, were acquired using Arccheck and evaluated with a 2%/2 mm gamma criterion.

**TABLE 1:** Measurements for beam model verification.

Measurements	Equipment	SSD (cm)	Depth (cm)	Field size (cm^2^)
PDD	IBA compact ionization chamber CC13 and diode detectors PFD, IBA Blue Phantom^2^ water tank	90		3 × 3, 5 × 5, 10 × 10, 30 × 30
Inline and crossline profiles	IBA compact ionization chamber CC13 and diode detectors PFD, IBA Blue Phantom^2^ water tank	90	5, 10, 20	3 × 3, 5 × 5, 10 × 10, 30 × 30
OF	IBA compact ionization chamber CC13 and diode detectors PFD, IBA Blue Phantom^2^ water tank	90	10	3 × 3, 5 × 5, 10 × 10, 20 × 20, 30 × 30
Absolute dose	IBA FC65‐GX ion chamber, IBA WP1D Phantom water tank	100	5, 10	5 × 5, 10 × 10, 20 × 20
Beam test field	Arccheck cylindrical diode array system			3 × 3, 5 × 5, 10 × 10, 25 × 25, OAVL, C, T

### 2.3 Clinical plan measurement

**FIGURE 1:**
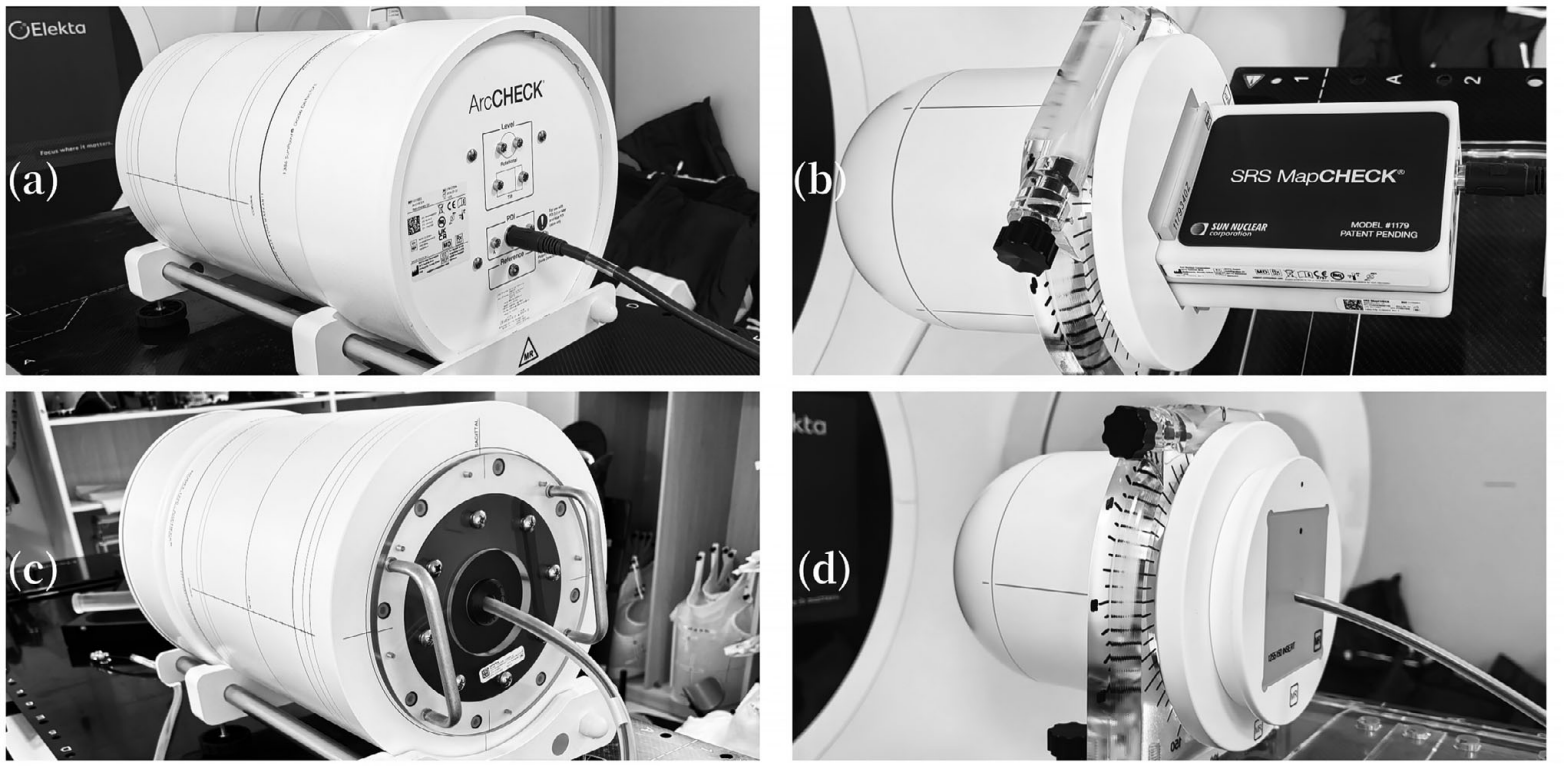
Measurement phantoms for CFRT and SBRT.Note: (a) and (b) represent the plan verification phantoms for CFRT and SBRT, respectively; (c) and (d) represents the absolute dose measurement phantoms for CFRT and SBRT, respectively.

The clinical plans of 31 patients (17 CFRT and 14 SBRT) involving different anatomical sites were selected for plan measurement on three linacs (two GBD linacs and one MBD linac). For CFRT cases, the cohort comprised three head‐and‐neck cases, six breast cases, two lung cases, one thymic case, and five rectal cases. The median patient age was 62 years (ranging between 48 and 70 years), with five males and 12 females. For each patient, volume‐modulated arc therapy (VMAT) plans were generated using 6MV photon beams in the Monaco TPS based on the GBD model. Plan verification and absolute dose measurement were subsequently performed using the Arccheck cylindrical diode array system (Sun Nuclear, Florida, USA) and the IBA FC65‐GX ion chamber (IBA Dosimetry, Schwarzenbruck, Germany). Plan verification results were assessed with a 3%/2 mm gamma criterion, and absolute dose measurement results were compared with TPS calculation results, respectively. For SBRT cases, the cohort comprised four brain metastasis cases, four lung cases, four liver cases, one adrenal tumor case, and one rib case. The median patient age was 67.5 years (ranging between 38 and 94 years), with ten males and four females. Lesion volumes ranged from 3.14 to 290.78 cm^3^. For each patient, VMAT plans were generated separately for 6MV FFF beams. Plan verification and absolute dose measurement were subsequently performed using SRS MapCHECK (Sun Nuclear, Florida, USA) and the CC01 ion chamber (IBA Dosimetry, Schwarzenbruck, Germany). Plan verification results were assessed with 3%/2 mm and 2%/2 mm gamma criteria, and absolute dose measurement results were compared with TPS calculation results, respectively. Details regarding measurement phantoms for both CFRT and SBRT are presented in Figure 1.

### 2.4 Statistical analysis

Statistical analyses were conducted using SPSS 26.0. Paired *t*‐tests were used to compare the gamma passing rates of the clinical plans on the three linacs. *P* < 0.05 was considered statistically significant.

## 3 RESULTS

### 3.1 Beam model verification

#### 3.1.1 PDD and profile verification

For all PDD and profile measurements using a 2%/2 mm gamma criterion, both GBD linacs against GBD achieved a 100% gamma passing rate for 6MV, 6MV FFF, and 10MV energies.

#### 3.1.2 OF verification

Table 2 shows comparisons of OF measurements acquired from the harmony pro‐GBD and infinity‐GBD linacs for various field sizes against GBD. For all field sizes, the agreement was within ± 1%. The largest deviation occurred for the 3 × 3 cm field. Specifically, for the Harmony Pro linac, differences in 6MV OFs ranged from −0.22 to 0.35%, those in 6MV FFF OFs ranged from −0.11 to 0.57%, and those in 10‐MV OFs ranged from 0.00 to 0.58%. Similarly, the Infinity linac showed deviations of −0.35 to 0.00% for 6‐MV OFs, −0.34 to 0.09% for 6‐MV FFF OFs, and −0.23 to 0.19% for 10‐MV OFs.

**TABLE 2:** Comparison of OF measurements from harmony pro‐GBD and infinity‐GBD against reference GBD values.

Energy	Flied (cm^2^)	Reference	Measurement	Difference (%)
6 MV	3 × 3	0.847	0.850^a^/0.844^b^	0.35^a^/‐0.35^b^
	5 × 5	0.907	0.905^a^/0.905^b^	−0.22^a^/‐0.22^b^
	10 × 10	1.000	1.000^a^/1.000^b^	0.00^a^/0.00^b^
	20 × 20	1.096	1.097^a^/1.096^b^	0.09^a^/0.00^b^
	30 × 30	1.143	1.145^a^/1.143^b^	0.17^a^/0.00^b^
6 MV FFF	3 × 3	0.874	0.879^a^/0.871^b^	0.57^a^/‐0.34^b^
	5 × 5	0.927	0.926^a^/0.925^b^	−0.11^a^/‐0.22^b^
	10 × 10	1.000	1.000^a^/1.000^b^	0.00^a^/0.00^b^
	20 × 20	1.064	1.064^a^/1.064^b^	0.00^a^/0.00^b^
	30 × 30	1.089	1.089^a^/1.090^b^	0.00^a^/0.09^b^
	3 × 3	0.861	0.866^a^/0.859^b^	0.58a/‐0.23^b^
	5 × 5	0.920	0.920^a^/0.920^b^	0.00^a^/0.00^b^
10 MV	10 × 10	1.000	1.000^a^/1.000^b^	0.00^a^/0.00^b^
	20 × 20	1.075	1.077^a^/1.077^b^	0.19^a^/0.19^b^
	30 × 30	1.110	1.113^a^/1.111^b^	0.27^a^/0.09^b^

*Note*: a refers to OF measurements from harmony pro‐GBD, and b refers to OF measurements from infinity‐GBD.

#### 3.1.3 Absolute dose verification

Table 3 presents a comparison between the measured absolute doses from both GDB linacs and the corresponding values calculated by the TPS. Across all field sizes and depths, measurements from both linacs remained within ±1.5% of the TPS calculations.

**TABLE 3:** Comparison of absolute dose measurements from harmony pro‐GBD and infinity‐GBD against calculations based on GBD.

		*d* = 5 cm			*d* = 10 cm		
Energy	Field (cm^2^)	Reference (cGy)	Measurement (cGy)	Difference (%)	Reference (cGy)	Measurement (cGy)	Difference (%)
6 MV	5 × 5	81.2	80.77^c^/80.72^d^	0.53^c^/0.59^d^	61.0	60.51^c^/60.70^d^	−0.80^c^/−0.49^d^
	10 × 10	86.5	86.60^c^/86.42^d^	0.12^c^/−0.09^d^	67.2	67.10^c^/67.16^d^	−0.15^c^/−0.06^d^
	20 × 20	92.2	91.70^c^/91.70^d^	−0.54^c^/−0.54^d^	73.6	73.31^c^/73.38^d^	−0.39^c^/−0.30^d^
6 MV FFF	5 × 5	82.9	82.57^c^/82.51^d^	−0.40^c^/−0.47^d^	62.0	61.55^c^/61.74^d^	−0.73^c^/−0.42^d^
	10 × 10	86.9	86.60^c^/86.63^d^	−0.35^c^/−0.31^d^	66.9	66.91^c^/66.82^d^	0.01^c^/‐0.12^d^
	20 × 20	90.5	90.91^c^/90.00^d^	0.45^c^/−0.55^d^	70.7	70.53^c^/71.00^d^	0.24^c^/0.42^d^
	5 × 5	85.1	85.57^c^/85.33^d^	0.550^c^/0.27^d^	68.2	68.57^c^/68.64^d^	0.54^c^/0.65^d^
10 MV	10 × 10	90.2	90.01^c^/90.40^d^	−0.21^c^/0.22^d^	72.0	72.59^c^/72.63^d^	0.82^c^/0.87^d^
	20 × 20	95.9	95.22^c^/95.30^d^	−0.71^c^/−0.63^d^	74.8	75.40^c^/75.62^d^	0.80^c^/1.10^d^

*Note*: c refers to absolute dose measurements from Harmony Pro‐GBD, and d refers to absolute dose measurements from Infinity‐GBD.

#### 3.1.4 Test field verification

Table 4 details the verification results for test field plans on the Harmony Pro‐GBD and Infinity‐GBD linacs. Using a 2%/2 mm criterion, all test fields achieved gamma passing rates above 98%.

**TABLE 4:** Test field verification results for harmony pro‐GBD and infinity‐GBD.

Energy	Field (cm^2^)	Harmony Pro‐GBD	Infinity‐GBD
6 MV	3 × 3	100.0	100.0
	5 × 5	100.0	100.0
	10 × 10	100.0	100.0
	25 × 25	98.1	100.0
	OVAL	100.0	100.0
	C	100.0	100.0
	T	99.0	98.9
6 MV FFF	3 × 3	100.0	100.0
	5 × 5	100.0	100.0
	10 × 10	100.0	100.0
	25 × 25	98.5	100.0
	OVAL	100.0	100.0
	C	100.0	100.0
	T	98.5	98.7
10 MV	3 × 3	100.0	100.0
	5 × 5	100.0	100.0
	10 × 10	100.0	100.0
	25 × 25	99.1	100.0
	OVAL	100.0	100.0
	C	100.0	100.0
	T	100.0	100.0

### 3.2 Clinical plan measurement

#### 3.2.1 CFRT plan

Table 5 summarizes the CFRT plan measurement results for the three linacs. The gamma passing rates under the 3%/2 mm criterion ranged from 96.5 to 99.7, 95.9 to 99.8, and 95.3 to 99.5% for the Harmony Pro‐GBD, Infinity‐GBD, and Infinity‐MBD linacs, respectively. All measured absolute dose deviations for the Harmony Pro‐GBD and Infinity‐GBD linacs were within 3%; for the Infinity‐MBD linac, all deviations were within 3% except for one (breast case: −3.73%). Except for this dose deviation, the test plans of the three linacs met clinical requirements.

**TABLE 5:** CFRT plan measurement results for the three linacs.

	Plan verification (%)	Dose discrepancy (%)
Harmony Pro‐GBD	98.07 ± 1.11	0.76 ± 1.07
Infinity‐GBD	98.53 ± 1.08	0.95 ± 1.11
Infinity‐MBD	97.61 ± 0.01	−0.95 ± 1.10
T	−1.674^e^/1.400^f^/2.459 ^g^	−1.333^e^/9.292^f^/7.805 ^g^
P	0.114^e^/0.181^f^/0.026 ^g^	0.201^e^/0.000^f^/0.000 ^g^

*Note*: e refers to the *t*‐ and *p*‐values for the comparison between harmony pro‐GBD and Infinity‐GBD, f refers to the *t*‐ and *p*‐values for the comparison between Harmony Pro‐GBD and Infinity‐MBD, and g refers to the *t*‐ and *p*‐values for the comparison between infinity‐GBD and infinity‐MBD.

#### 3.2.2 SBRT plan

Table 6 summarizes the SBRT plan measurement results for the three linacs. The Gamma passing rates of the Harmony Pro‐GBD and Infinity‐GBD under the 3%/2 mm and 2%/2 mm criteria exceeded 95 and 90%, respectively, with absolute dose deviations within 3%. For the Infinity‐MBD, the range of gamma passing rates under the 3%/2 mm criterion were between 88.4 and 100%, with two cases below 90% (88.4 and 89.0%); under the 2%/2 mm criterion, the range was between 81.1 and 96.9%. Absolute dose deviations exceeded 3% in two cases (lung case: −3.51%, brain metastasis case: −4.50%), while the remaining deviations were within 3%. Both the plans of the Harmony Pro‐GBD and Infinity‐GBD linacs met clinical requirements, while those of the Infinity‐MBD linac met clinical requirements in most, but not all, cases.

**TABLE 6:** SBRT plan measurement results for the three linacs.

	Plan verification (%)		
	3%/2 mm	2%/2 mm	Dose discrepancy (%)
Harmony Pro‐GBD	98.18 ± 1.58	96.69 ± 1.96	−0.14 ± 1.30
Infinity‐GBD	98.76 ± 1.54	96.29 ± 2.26	0.52 ± 1.22
Infinity‐MBD	94.72 ± 0.04	89.51 ± 0.06	−0.91 ± 1.68
T	−1.837 ^h^/3.316^i^/4.029^j^	0.661 ^h^/4.988^i^/4.156^j^	−1.703 ^h^/2.69^i^/4.371^j^
P	0.089 ^h^/0.006^i^/0.001^j^	0.520 ^h^/0.000^i^/0.000^j^	0.112 ^h^/0.019^i^/0.001^j^

*Note*: h refers to the *t*‐ and *p*‐values for the comparison between Harmony Pro‐GBD and Infinity‐GBD, i refers to the *t*‐ and *p*‐values for the comparison between Harmony Pro‐GBD and Infinity‐MBD, and j refers to the t‐ and P‐values for the comparison between Infinity‐GBD and Infinity‐MBD.

## 4 DISCUSSION

Traditionally, linac commissioning, data acquisition, and TPS beam modeling have been complex and labor‐intensive processes that require extensive, high‐precision measurements, placing substantial demands on the expertise and skill of medical physicists. These tasks have long been recognized as both challenging and time‐consuming.^11^, ^12^, ^13^ The AGL workflow, based on GBD, addresses these challenges by streamlining key steps such as data acquisition and beam modeling, thereby notably enhancing efficiency while reducing dose discrepancies arising from modeling errors. This study aimed to verify the performance of GBD on two different Elekta linac models, namely Harmony Pro and Infinity, to provide a practical reference for the clinical application of GBD.

During the commissioning of both the Harmony Pro and Infinity linacs, each task was performed by one to two specialized personnel, with an average daily working time of approximately eight hours. By adopting the AGL workflow, the overall acceptance and commissioning period was reduced from the conventional eight weeks to six weeks, which marked an efficiency improvement of 33.33%, thereby expediting clinical deployment. Furthermore, analysis of beam model accuracy demonstrated that, for both the Harmony Pro and Infinity linacs, the GBD model yielded exceptionally high agreement with measurements. Specifically, all PDD and profile measurements achieved 100% gamma passing rates under a 2%/2 mm gamma criterion, while differences in OF measurements remained within ± 1%. These findings are consistent with those reported by Kannan et al.^14^ in their study of three Versa HD beam modeled using GBD. This high level of agreement can be primarily attributed to the high‐precision control exercised during BM. Specifically, while the vendor tolerance for BM is set at 1%, the BM errors between the Harmony Pro and Infinity linacs in this study were maintained within 0.5%. Notably, in this study, gamma analysis was employed to assess the consistency between the measured PDD and profiles and the GBD reference data. Although the gamma index effectively evaluates overall agreement, it has inherent limitations.^15^, ^16^, ^17^ Additionally, because the gamma value is an absolute metric that always remains positive, it cannot convey the direction of dose deviations when failures occur.^18^

The American Association of Physicists in Medicine (AAPM) Medical Physics Practice Guidelines state that, to ensure beam accuracy, one should not rely solely on matching the measured beam data of each individual linac to reference values. Instead, calculations based on the TPS physical model should also be incorporated as a benchmark, as this approach more directly evaluates discrepancies between the performance of the linac and the expected output of the TPS.^19^, ^20^ To enhance data reliability, we followed this recommendation by comparing absolute dose and test field measurements against corresponding calculations from the TPS. Similarly, the results demonstrated high consistency. Specifically, differences in absolute doses remained within ±1.5%, while the gamma passing rates for all test fields exceeded 98% under a 2%/2 mm gamma criterion, further confirming the superior agreement between TPS‐calculated and measured dose distributions. Collectively, these verification results indicate that GBD are capable of accurately capturing the dosimetric characteristics of different linac models, providing a reliable basis for clinical dose calculations.

To further validate the clinical applicability of the GBD model, we selected actual clinical plans from different anatomical sites for testing and introduced an additional Infinity linac using MBD modeling. By verifying the gamma passing rates and absolute dose deviations across the three linacs, we compared their dosimetric differences to evaluate whether Monaco TPS plans based on the GBD model can meet clinical requirements. Seventeen CFRT plans involving different anatomical sites were selected for plan measurement. Gamma passing rates on both GBD linacs exceeded 95% under the 3%/2 mm gamma criterion for all plans, with dose discrepancy remained within ±3%. This outcome aligns with the findings of Li et al.,^8^ who performed three‐dimensional dose verification using VMAT for three Versa HD linacs (Synergy1, Synergy2, and VersaHD) modeled using GBD. They found that all VMAT plans achieved gamma passing rates over 95%, well within clinical tolerances. For the CFRT Infinity‐MBD linac, all 17 tested verification plans achieved gamma passing rates (3%/2 mm) above 95%, with no statistically significant differences from the Harmony Pro‐GBD. Only one breast case showed an absolute dose deviation exceeding the clinically acceptable threshold (>3%), while the remainder of cases met clinical requirements. This demonstrates that the 6‐MV X‐ray beam of this MBD‐modeled linac closely approximates the gold‐standard beam quality, validating the GBD model's applicability to MBD‐modeled linacs. The results also highlight that relying solely on gamma passing rates may not fully guarantee the accuracy of clinical dose delivery. Absolute dose measurements have to be combined for dual verification, especially when accelerator interchange is required during patient treatment. In the AGL workflow based on GBD, the minimum measured field size is 3 × 3 cm. This field size exhibited the largest deviation in OF values. However, in CFRT plans, the impact of small‐field OF discrepancies on overall dose distribution is difficult to detect using standard verification methods. This difficulty arises because small fields contribute minimally to the total dose, and detector systems within the verification equipment have inherent sensitivity limitations. For SBRT plans, dose distributions result from accumulated small fields. Therefore, the accuracy of small‐field dosimetry directly influences the overall dosimetric accuracy. Thus, for linacs employing the AGL workflow, routine testing of CFRT plans alone is insufficient for implementing stereotactic techniques. Specific testing of SBRT plans is also necessary. Scholars have emphasized that dosimetric precision is crucial when transferring SBRT patients between linacs. Extreme caution must be exercised when transitioning patients to beam‐matched linacs to ensure treatment accuracy.^21^, ^22^ For this reason, we tested SBRT plans using the small‐field verification device SRS MapCHECK, adopting a more stringent gamma analysis criterion of 2%/2 mm. Our results indicated gamma passing rates above 95 and 90% for both GBD linacs under the 3%/2 mm and 2%/2 mm criteria, respectively. Additionally, absolute dose deviations were within 3%, with no statistically significant differences. Xu et al.^23^ performed similar tests on SBRT plans using ArcCHECK and Gafchromic EBT3 films for three beam‐matched linacs, reporting findings consistent with this study. For the SBRT Infinity‐MBD linac, two plans exhibited a gamma passing rate below 90% under the 3%/2 mm criterion; moreover, two plans demonstrated an absolute dose deviation exceeding 3% (lung case: −3.51%, brain metastasis case: −4.50%). Although the remaining cases met clinical requirements, the overall validation results (gamma passing rate and dose deviation) were inferior to those from the two GBD linacs. On the one hand, these findings confirm the clinical applicability of the GBD model in the Monaco TPS. On the other hand, they clearly indicate that the Monaco TPS (equipped with the MBD model) should be employed for plan design to ensure treatment accuracy for patients undergoing SBRT on the Infinity‐MBD linac.

In this study, beam measurements from two GBD linac models—Harmony Pro and Infinity—showed excellent agreement with GBD. This agreement is primarily attributed to two factors. First, during BM, both linacs were tuned to match the GBD using a tighter 0.5% tolerance. Prior investigations have indicated that the vendor's default 1% tolerance is overly permissive and, therefore, have recommended reducing the tolerance to 0.5%, particularly for linacs intended for stereotactic treatments.^24^, ^25^ Second, both GBD linacs had been in clinical use for less than one year, experienced few mechanical issues, and had not undergone any major component replacements, indicating that they remained in a relatively stable state. Therefore, ongoing monitoring of beam stability for both GBD linacs is essential to guarantee the interchangeability of treatment delivery during patient care. For linacs employing GBD for modeling, a more rigorous quality control protocol is recommended to maintain beam reproducibility. Nevertheless, this study has certain limitations. Specifically, verifications were performed only for the two most commonly used photon energies—6MV, 6MV FFF and 10MV. Therefore, our conclusions should not be directly extrapolated to other energy settings. Future research is warranted to evaluate the performance of GBD at additional energies and with other beam modalities. We employed only one Infinity MBD‐model linac as the control group; future research should expand the sample size by including additional MBD‐model linacs (e.g., Harmony Pro, Synergy, Versa HD) for systematic comparison with GBD‐model linacs, thereby enabling a more comprehensive evaluation of the value and reliability of GBD technology.

## 5 CONCLUSION

Our results demonstrate that the Elekta GBD‐based beam modeling approach is clinically feasible for the Harmony Pro and Infinity linacs. The Monaco TPS plan design based on the GBD model did not only meet the clinical requirements of linacs using gold‐standard beam data modeling but is also applicable to the majority of linacs employing conventional measurement datasets for modeling. This demonstrates that the GBD model in the Monaco TPS can be reliably applied in clinical settings. By using the GBD, a beam model that ensures consistent and accurate dose calculation can be rapidly and accurately established, thereby providing essential technical support for clinical radiotherapy treatment. Despite some limitations, the findings of this study hold important clinical relevance. As radiotherapy technology evolves, GBD is expected to play an increasingly important role in improving treatment quality and streamlining clinical workflows.

## AUTHOR CONTRIBUTIONS

Min Zhang, Wenchao Gao, and Shuchao Zhang designed the study. Wenchao Gao, Jie Qi, Xin Wang, Liang Zhao, Ping Liu, Zhijun Yang, and HongXiang Cheng participated in the data collection. Wenchao Gao and Shuchao Zhang carried out the data analysis, while Min Zhang and Wenchao Gao wrote the manuscript. All authors contributed to critical manuscript revisions and approved the final version for submission. Min Zhang bears the ultimate responsibility for the decision to submit the manuscript for publication.

## CONFLICT OF INTEREST STATEMENT

The authors declare no conflicts of interest.

## ETHICS STATEMENT

This study was approved by the Ethics Committee of Peking University People's Hospital (Approval No. 2025PHB428‐001).

## Data Availability

Research data are not available at this time.
